# Cam-Unet: Print-Cam Image Correction for Zero-Bit Fourier Image Watermarking

**DOI:** 10.3390/s24113400

**Published:** 2024-05-25

**Authors:** Said Boujerfaoui, Hassan Douzi, Rachid Harba, Frédéric Ros

**Affiliations:** 1 IRF-SIC, University of Ibn Zohr, Agadir 80000, Morocco; 2 PRISME, University of Orléans, 45000 Orléans, France

**Keywords:** image watermarking, neural networks, print-cam watermarking, Fourier transform, geometric distortions, robustness, digital images, deep learning

## Abstract

Image watermarking often involves the use of handheld devices under non-structured conditions for authentication purposes, particularly in the print-cam process where smartphone cameras are used to capture watermarked printed images. However, these images frequently suffer from perspective distortions, making them unsuitable for automated information detection. To address this issue, Cam-Unet, an end-to-end neural network architecture, is presented to predict the mapping from distorted images to rectified ones, specifically tailored for print-cam challenges applied to ID images. Given the limited availability of large-scale real datasets containing ground truth distortions, we created an extensive synthetic dataset by subjecting undistorted images to print-cam attacks. The proposed network is trained on this dataset, using various data augmentation techniques to improve its generalization capabilities. Accordingly, this paper presents an image watermarking system for the print-cam process. The approach combines Fourier transform-based watermarking with Cam-Unet as perspective distortion correction. Results show that the proposed method outperforms existing watermarking approaches typically employed to counter print-cam attacks and achieves an optimal balance between efficiency and cost-effectiveness.

## 1. Introduction

The evolution of networking technologies has enabled various mechanisms for efficient data transfer and exchange, enabling the seamless movement of high information volumes with exceptional efficiency and speed [1]. At the same time, this evolution increases the potential surface for exploitation. As data traverses across networks, platforms, and devices, data become susceptible to interception, manipulation, and corruption. Given these challenges, industries are currently faced with the urgent need to rapidly explore real-time solutions that can effectively promote secure data processing. In recent years, watermarking has become a fast-growing field of research, attracting considerable interest from the scientific community [2]. Although relatively new, watermarking has proved to be a promising solution in various security fields, such as authentication and copyright protection.

Image watermarking involves embedding copyright information or verification messages, known as watermarks, into digital images. The watermark typically consists of a pattern of bits, and its content varies according to the target application. The embedding process must ensure that the watermark does not significantly degrade the quality of the image and remains imperceptible. At the same time, the watermark should be sufficiently robust to resist various forms of attack or manipulation that the digital image may encounter during transmission or unauthorized use [3]. Once the watermarked image is transmitted over a communication channel and reaches its recipient, the embedded watermark must be detected. The detection process needs to be reliable, accurate, and capable of identifying the watermark even if the image has undergone multiple distortions.

**Figure 1:**
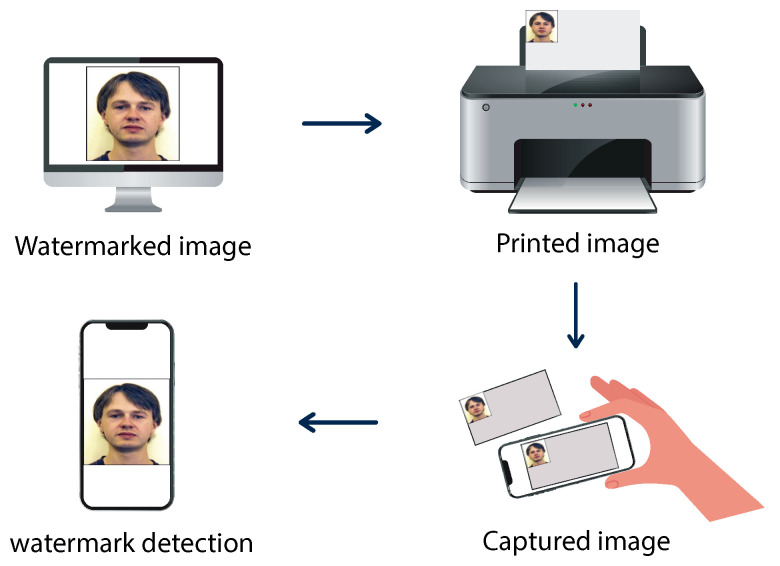
The process of print-cam image watermarking.

Recently, image watermarking algorithms have been adapted for mobile device systems, enabling real-time authentication solutions accessible on smartphones. In this study, we focused on print-cam watermarking [4], a process of embedding watermarks into an image that is intended for printing on physical media such as paper or plastic cards. This process involves capturing the printed image using a smartphone camera in a freehand manner, facilitating the identification of hidden information. However, several challenges arise in this scenario, primarily stemming from perspective transformations and alignment issues when capturing images from different viewpoint angles. These challenges can affect the accuracy of watermark detection [5], as changes in perspective can distort the original watermark pattern, making it difficult to detect accurately. Figure 1 shows the print-cam watermarking process.

Image watermarking techniques have already seen significant developments, leveraging various domains such as spatial and frequency domains [6]. With the emergence of deep learning, there has been a notable shift towards utilizing neural networks for watermarking tasks due to their ability to extract complex features from images, leading to more efficient and robust watermarking methods [7]. However, despite these advancements, no single watermarking technique can provide a complete defense against all possible attacks. Each technique often serves specific purposes and comes with its own set of advantages and limitations. For instance, some methods may excel in robustness against common image processing manipulations like noise addition or compression [8], while others may prioritize imperceptibility or resistance against geometric distortions [9,10].

Image watermarking techniques face particular challenges in scenarios involving the print-cam process, which introduces perspective distortions [11]. These distortions result from the combined effects of rotation, translation, scaling (RST), and angular tilt of the optical axis between the image and the camera. Despite the importance of addressing projective distortions in print-cam watermarking, research in this area remains relatively sparse. The complexity of these distortions and their impact on watermark robustness demand specialized attention. Consequently, there is a need for further exploration and development of robust watermarking algorithms tailored specifically for print-cam scenarios. To address the challenges posed by projective distortions, researchers may explore innovative approaches that combine traditional watermarking techniques with advancements in computer vision and geometric transformation modeling. Additionally, leveraging deep learning methodologies could prove beneficial in learning representations robust to these distortions, thereby enhancing the effectiveness of watermarking algorithms in print-cam environments.

This paper presents Cam-Unet, a novel learning-based method to rectify print-cam distorted images. Previous approaches employed learning solely for feature extraction, whereas the final image recovery still depended on traditional optimization practices. Instead, our method directly predicts image distortion using convolutional neural networks (CNNs). By leveraging CNNs for end-to-end image retrieval, the method avoids the need for complex optimization procedures, which not only simplifies the process but also improves efficiency during testing. Cam-Unet is trained to predict the forward mapping that moves pixels from a distorted image $I_{D}$ to their corresponding positions in a rectified result image $I_{R}$. This approach can be seen as analogous to semantic segmentation, where instead of assigning class labels to pixels, the network predicts 2D vectors representing the displacement of each pixel. To effectively train the network, a large amount of images is required along with corresponding deformations. At present, no dataset meeting these criteria exists, and obtaining ground truth deformations from the real-world environment is exceedingly difficult. Consequently, we opt to generate synthetic data for training purposes. We create a dataset of 100k images by randomly distorting the flat images. In this process, the distorted image serves as the input, while the mesh utilized to deform the image represents the inverse deformation that we aim to recover through training.

The final goal of this paper is to design an image watermarking technique that can withstand print-cam attacks for images captured manually using a mobile device camera. To achieve this, we combine the Fourier-based method [12] for watermark embedding with Cam-Unet for image correction. The Fourier-based method was chosen due to its proven resistance against geometric distortions [13], which are commonly encountered in the print-cam process. We conducted experimental tests to assess the effectiveness of our approach. In the first test, we subjected framed ID images to simulated perspective attacks, while in the second test, we exposed them to real print-cam attacks using two distinct smartphones: Redmi Note 10 and Samsung S21. Subsequently, we compared the performance of our proposed method in terms of robustness and efficiency against existing competitive methods.

The main contributions of this work are as follows:

1. The first end-to-end learning-based method to directly rectify print-cam images. This network is trained to map the distorted images to their rectified states;
2. An innovative methodology to synthesize print-cam images along with corresponding distortion maps, accurately simulating real-world printing and capturing conditions;
3. Improvement of Fourier watermarking for the print-cam process through integration with Cam-Unet for image rectification. This fusion technique showcases superior performance compared to existing methods;

The article is organized as follows: Section 2 reviews the state-of-the-art techniques in print-cam watermarking. In Section 3, we present the overall watermarking system, including the new correction method. Section 4 showcases experimental results achieved in simulated and real-world scenarios. Finally, Section 5 concludes the paper.

## 2. Related Works

Watermarking techniques for digital images typically fall into two main types: spatial domain and transform domain methods [14]. In spatial domain approaches, watermarks are inserted by directly modifying the pixel matrices of images, while transform domain techniques involve embedding watermarks in the frequency spectrum. Some methods combine both domains to leverage their respective advantages [15]. The choice between these domains depends on various factors such as the desired level of robustness, imperceptibility, computational complexity, and specific application requirements [16].

Industrial watermarking encounters a major challenge during the print-cam process, attributed to complex geometric distortions such as rotation, scale, translation (RST), and the effects of camera tilt. To address this synchronization challenge, several strategies have emerged in the existing literature. Nakamura et al. [17] and Katayama et al. [18] conducted one of the first studies to confirm the need for watermarking approaches in the print-cam process. They introduced a spatial domain watermarking technique for camera-equipped mobile phones, designed to withstand geometric distortions through frame synchronization. However, their approach was limited in terms of robustness to small geometric distortions. Takeuchi et al. [19] used frame synchronization with guided scrambling techniques to overcome geometric distortions, resulting in an improved performance against lens distortion. Nonetheless, it could withstand minor geometric distortions. Kim et al. [20] introduced a spatial domain embedding technique. Their approach involved using a spatial template to predict geometric deformations, leading to successful watermark detection. Notably, they employed a tripod during the capture process to minimize geometric attacks. Liu et Shieh [21] suggested refining the watermarking approach used by Nakamura et al. [17]. Their proposal involved using the Watson model to calculate the Just Noticeable Difference (JND) and adjust the strength of the embedded mark accordingly. This refinement not only strengthened resilience but also enhanced invisibility.

Pramila et al. [22] first used the term print-cam process when they proposed a watermarking technique based on discrete wavelet transform (DWT). Their methodology incorporates a visible framework for 3D synchronization, leading to enhanced outcomes in terms of resilience against geometric distortions. However, these results are relatively attributed to the careful capture process involved. After that, Pramila et al. [23] applied an autocorrelation function to determine the alignment of the pseudorandom pattern and read the multi-bit watermark message for the print-cam process. Tailored for color images and omitting the need for frames, this approach addresses the challenge of unfocused images in the capture process. Thongkor and Amornraksa [24] proposed a digital image watermarking system for photo authentication on ID cards. Their approach involved the use of a spatial domain watermarking method to embed the watermark specifically in the blue color channel of the image. To rectify geometric distortions, they used the original image. Despite obtaining positive results, this method worked as a non-blind approach. In a subsequent study, Thongkor and Amornraksa [25] suggested enhancing the preceding method by integrating a modified JND model and an automatic image registration to reduce perspective distortions. However, this approach still requires the original image for watermark detection.

Spatial and wavelet transform watermarking techniques are commonly preferred over Fourier transform methods for the print-cam process. However, Fourier-based watermarking methods are recognized for their robustness against geometric transformations, excluding changes in scale, and optical axis tilting distortions. Khadija et al. [26] introduced a robust print-cam image watermarking method based on the Discrete Fourier Transform (DFT) tailored for ID images. This approach implemented perspective correction using the Hough lines transform before the detection stage, which effectively mitigated geometric distortions and produced promising outcomes. Afterward, Boujerfaoui et al. [27] integrated a neural network architecture into the Fourier-based framework [26] to improve perspective correction capabilities. By leveraging the neural network, the method achieved superior resilience against print-cam attacks compared to its predecessor, improving the overall robustness of the watermarking technique in practical scenarios.

Deep learning techniques, specifically convolutional neural networks (CNNs) [28], have been widely employed in numerous studies to automate the watermarking process and subsequent extraction. CNNs excel in learning correlations between watermarked and original images, striking a balance between robustness to distortions and image quality preservation. Most learning-based methods employ auto-encoders for image watermarking, as they have demonstrated the ability to hide the watermark within the invisible perturbations of the digital image [29]. In addition, approaches addressing synchronization challenges often incorporate distortion layers during training to effectively handle these issues and enhance the overall performance of watermarking systems [30,31]. However, the trained model may not efficiently handle unseen attacks.

In the context of print-cam watermarking, the number of learning-based methods proposed in the literature is still relatively limited compared to conventional methods. Tancik et al. [32] introduced StegaStamp, a steganographic algorithm tailored for encoding and decoding hyperlink bit-strings within print-cam images. This method involves rectifying watermarked images using a fine-tuned technique [33] to segment potentially marked regions. While the prototype system demonstrates reliability against print-cam attacks, there remains a need to enhance the visual quality of watermarked images. Qin et al. [34] presented an end-to-end network architecture designed for both watermark embedding and extraction processes. They employed a deep noise simulation network with a multitask loss function to produce highly robust watermarked images. Notably, their focus was mainly on addressing the noise attack in print-cam scenarios.

In contrast, significant contributions have been tailored to the screen-cam process. In this scenario, rather than printing the watermarked images, they are displayed on a computer screen and captured using a smartphone camera. This process is susceptible to similar attacks as those encountered in the print-cam process. Zhong et al. [35] introduced an unsupervised blind technique using deep neural networks for robust and secure watermark embedding and extraction. Their approach integrates random encoding and invariance layers to mitigate attacks and adjust to different camera scenarios. However, it encounters challenges related to data processing speed and geometric attacks. Additionally, Jia et al. [36] proposed a 3D rendering transformation network to enhance robustness, while using a JND-based loss function during training to maintain the visual attraction of the image. In a recent study, Liu et al. [37] presented a watermarking system to resist camera-based attacks, focusing on automatic watermark localization and detection. This method achieves reliable automatic extraction, but the detection accuracy and image transparency are affected by the embedding intensity.

In summary, print-cam image watermarking has undergone significant development, driven by various contributions aimed at preserving image content, whether using conventional or learning-based methods [38]. However, despite these advancements, no single method exists that can fully withstand the perspective distortions inherent in print-cam scenarios. We find that the main reason for this challenge lies in the instability of perspective correction results. Therefore, enhancing these results represents a critical issue. By combining the capabilities of deep learning with conventional watermarking methods, we aim to mitigate the challenges posed by print-cam perspective distortions and provide a more resilient solution.

## 3. Proposed Print-Cam Watermarking Method

**Figure 2:**
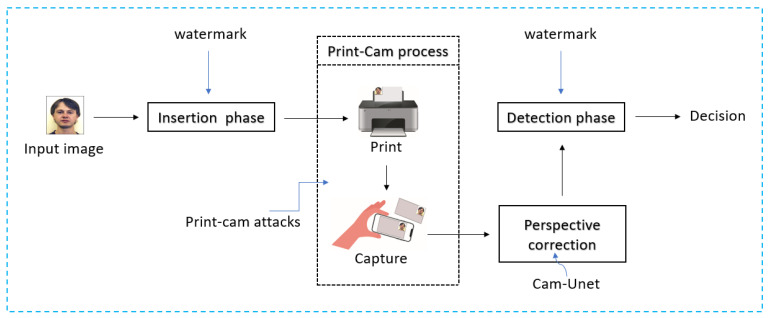
The proposed print-cam watermarking design.

The watermark is initially inserted into the cover image. Following the print-cam process, the captured image undergoes perspective correction. Subsequently, during the detection stage, a decision is made regarding whether the rectified image contains the watermark or not. The proposed watermarking method is illustrated in Figure 2.

### 3.1. Fourier Watermarking Method

#### 3.1.1. Watermark Insertion

The watermark is inserted into the magnitude of the Discrete Fourier Transform (DFT) along a circular region of radius r. In this process, only the luminance components of the color image are used, while the chrominance components remain unmodified. Then, a dedicated low-pass filter is applied to the embeddable coefficients of the DFT magnitude. According to [12], this filtering process helps in enhancing the detection rate. Thus, the watermark W is embedded into the filtered coefficients using the formula:

(1) $$M_{W}=M_{f}+\alpha \times W$$

where $M_{W}$ represents the magnitude of the watermarked coefficients, $M_{f}$ refers to the original coefficients after applying a Gaussian filter to the embeddable coefficients, and α is the insertion strength. The algorithm iteratively adjusts α to attain a specific PSNR for the watermarked image. Using a binary search within a defined range $\left[\alpha_{min},\alpha_{max}\right]$, the algorithm dynamically updates α by halving the search interval in each iteration, thereby efficiently narrowing down the possible range where the optimal alpha lies. This process continues until the calculated PSNR closely matches the target PSNR of 40 dB with an error of 0.001 [39]. Notably, The watermark length is limited to 180 bits. The final watermarked image is subsequently reconstructed, which entails applying the inverse Discrete Fourier Transform (IDFT) to retrieve the luminance of the watermarked image. The unmodified chrominance components are then combined with the watermarked luminance to reconstruct the final color image.

#### 3.1.2. Watermark Detection

The detection process remains blind as the decoder solely depends on the watermarked image and the watermark, without requiring the original or non-watermarked image. Initially, it applies the DFT algorithm to the luminance of the watermarked image. Next, it extracts the coefficients positioned along the radius r, corresponding to the circular region where the watermark was originally embedded during the encoding phase. The maximum normalized cross-correlation, $C_{max}$, is calculated between the extracted Fourier coefficients *F* and the watermark W.

(2) $$C_{max}=\underset{0\leq j\leq 1}{max}\left(\frac{\sum_{i=0}^{N-1}\left(W\left(i\right)-\bar{W}\right)\left(F\left(i+j\right)-\bar{F}\right)}{\sqrt{\sum_{i=0}^{N-1}{\left(W\left(i\right)-\bar{W}\right)}^{2}\sum_{i=0}^{N-1}{\left(F\left(i+j\right)-\bar{F}\right)}^{2}}}\right)$$

Here, the mean of the watermark sequence is denoted as $\bar{W}$, while $\bar{F}$ represents the mean of the extracted coefficients, with N indicating the length of the watermark. The watermark is considered present if $C_{max}$ exceeds a predefined threshold t. The value of t is chosen to minimize the probability of false alarm (PFA), also known as the Neyman–Pearson criterion [40]. In the context of Fourier watermarking, a theoretical model is used to calculate the PFA for a given threshold value t, as shown below [41]:

(3) $$P_{\mathrm{fa}}=P\left(C>t\right)=\frac{\int_{0}^{\cos^{-1}\left(t\right)}\sin^{L-2}\left(u\right)\;du}{2\int_{0}^{\frac{\pi }{2}}\sin^{L-2}\left(u\right)\;du}$$

where *C* represents the value of the cross-correlation coefficient, while *L* denotes the length of the watermark.

### 3.2. Print-Cam Perspective Correction

To counteract perspective attacks during the print-cam process, a novel learning-based correction approach called Cam-Unet has been developed.

#### 3.2.1. Cam-Unet Architecture

**Figure 3:**
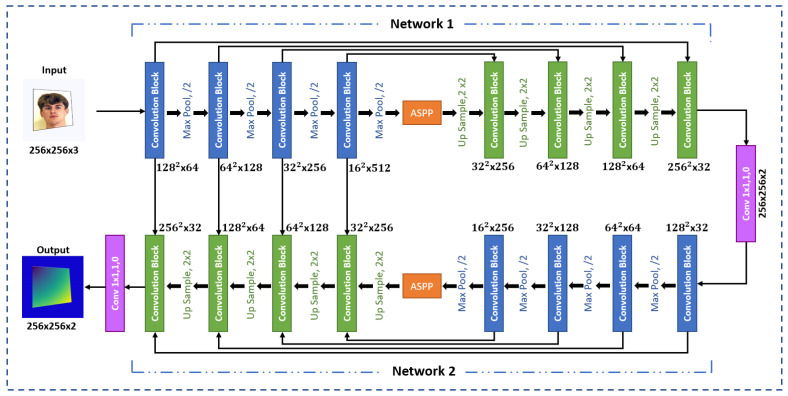
Cam-Unet architecture.

Figure 3 provides an overview of the proposed architecture. Cam-Unet integrates the components of Dual U-Net [42], attention mechanisms, and Atrous Spatial Pyramid Pooling (ASPP) modules [43].

The U-Net architecture comprises an analysis path (encoder) with convolutional blocks and max-pooling layers for feature extraction and dimensionality reduction, as well as a synthesis path (decoder) with up-convolutional layers and skip connections for feature refinement and spatial upsampling. Notably, U-Net uses skip connections to link the feature maps from the analysis path to the corresponding ones in the synthesis path, enabling the network to retain fine details and multi-scale information. Thus, adding additional layers to the U-Net architecture will enable the network to learn more representative features, leading to better output predictions. From this perspective, the ASPP module is used as an intermediary between the encoder and decoder sub-networks to capture multi-scale contextual information.

**Figure 4:**
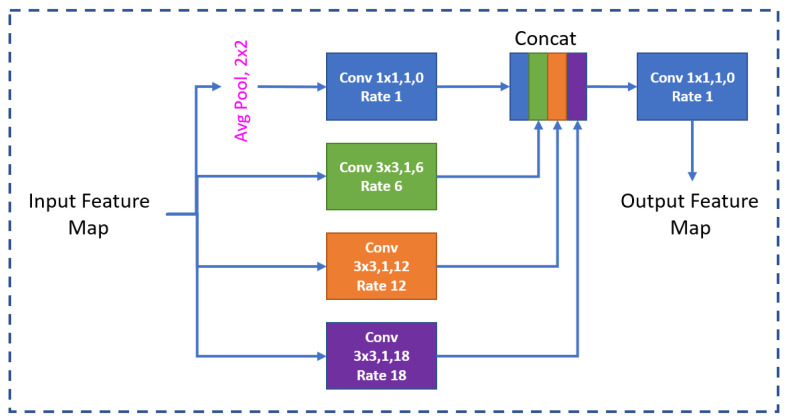
ASPP module.

The ASPP module, illustrated in Figure 4, includes an adaptive average pooling layer followed by convolutional layers with different dilation rates and a final convolutional layer to fuse the features. This helps to accurately predict pixel-wise labels, particularly in complex scenes where data can vary considerably in size and shape. Consequently, by aggregating information from multiple scales and spatial locations, the ASPP module enriches the feature representation, which is then passed on to the decoder.

In the view of Figure 3, the architecture consists of two main networks, labeled Network 1 and Network 2. Each network follows the same structure, starting with an encoder sub-network (marked in blue) followed by a decoder sub-network (marked in green). In the first network (Network 1), a three-channel input image is initially processed by the encoder. The resulting output is then subjected to further processing through the ASPP module. Following this, the decoder concatenates the output from the ASPP module with skip connections from the encoder. In the second network (Network 2), the encoder operates on a 2-channel input derived from the previous network (Network 1). Afterward, The encoder output is fed into the ASPP module. The decoder then concatenates the output from the ASPP module with skip connections from both encoders. Finally, the prediction yields an output with two channels.

**Figure 5:**
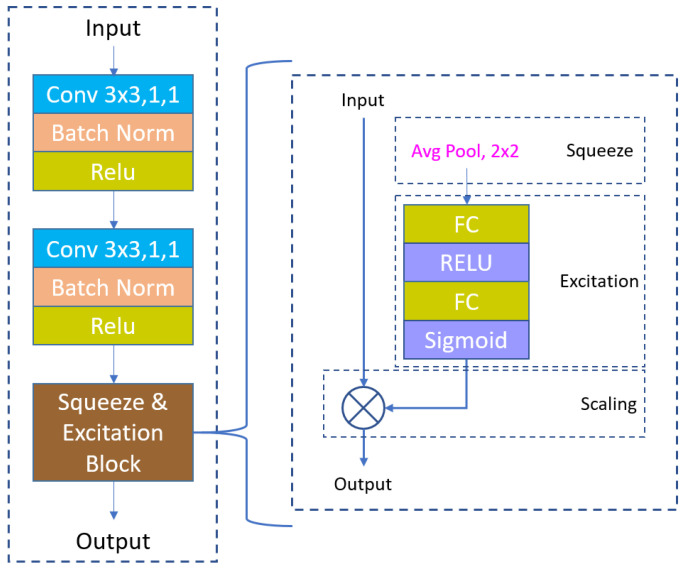
Convolution block.

Figure 5 shows the design of the convolution block used in the architecture. Each block performs two 3 × 3 convolution operations, followed by a batch normalization to reduce internal covariate shift and regularize the model. Subsequently, a rectified linear unit (ReLU) activation function is employed to introduce non-linearity. The squeeze-and-excitation block, applied after the convolutional layers, modulates channel-wise responses to highlight informative features. Notably, max-pooling is conducted with a 2 × 2 window and stride of 2 after each encoder convolution block, while a 2 × 2 bi-linear upsampling operation is performed before each decoder convolution block. The first decoder employs skip connections from the convolutional blocks of the first encoder, whereas the second decoder integrates skip connections from the convolutional blocks of both encoders to maintain spatial resolution and improve the quality of the output feature maps. At the end of the architecture, a convolution layer is applied to generate the final output prediction.

We assume that the produced forward map from the first network can be enhanced and refined with the assistance of the second network [44], leading to more accurate and improved predictions. This approach is the primary motivation behind the use of two U-Net networks. The squeeze-and-excite block between the inside of the convolutional blocks serves to reduce redundant information while preserving the most pertinent features. The adoption of ASPP modules contributes to the extraction of high-resolution feature maps, leading to enhanced performance. Also, the skip connections between the encoder of Network 1 and the decoder of Network 2 preserve fine-grained spatial information that might otherwise be lost during down-sampling operations. These connections enable the fusion of features from multiple scales, allowing the network to understand complex spatial structures in the image.

The proposed architecture takes a distorted image of dimensions h × w × 3 and outputs a 2D coordinate map. Instead of predicting discrete class labels, the network is designed to generate continuous coordinate values representing the precise location of each pixel in the rectified image. Consequently, every pixel in the input image is associated with specific (x, y) coordinates in the output map, essentially defining its position in a flattened space. By adopting this regression-based approach, the model achieves a finer and more accurate mapping between distorted and rectified images compared to the commonly used pixel-wise classification methods in semantic segmentation.

#### 3.2.2. Dataset Preparation

Overall, training convolutional neural networks (CNNs) with print-cam images poses challenges in terms of data collection and representation. Innovative approaches such as synthetic data generation, data augmentation, and transfer learning can help overcome these challenges and improve the robustness and accuracy of trained models.

We plan to use synthetic data for training, a common practice observed in recent deep-learning systems [45,46]. The approach involves directly creating training images in 2D, which can be more accessible and computationally efficient compared to manipulating 3D meshes. This enables faster image generation and allows for greater control over the dataset generation process. Furthermore, synthetic data generation makes it possible to create deformations covering a wide range of scenarios, including those that may be difficult to capture in real-world data. In this work, the main objective is to map from a distorted image to its rectified state. In contrast, the data synthesis process is entirely inverse to this procedure.

**Figure 6:**
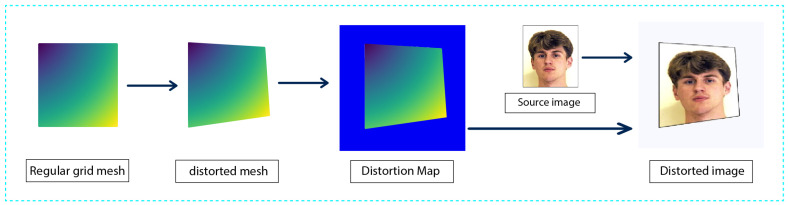
Data synthesis process. The network takes the distorted image as input, with the distortion map as ground truth.

Our main application concerns print-cam watermarking of ID images. Accordingly, we subject ID images sourced from the PICS database [47] to random distortions similar to those that occur when capturing images with a smartphone. Figure 6 visualizes the process of data synthesis.

For a given image *I*, An m×n mesh *M* is created to serve as control points for the deformation process. The initial deformation point *p* is randomly selected from the vertices of *M*. This point serves as the starting point of the deformation. The deformation direction and magnitude, denoted as *v*, are randomly generated. The deformation direction indicates the direction in which each vertex of the mesh will be displaced, while the magnitude of deformation determines the extent to which each vertex will be displaced. The resulting deformed mesh is calculated by adding the deformation vector *v* to each vertex $p_{i}$ of the mesh *M*, resulting in a new vertex position $(p_{i}+v),\forall i$. The sparse deformation field is interpolated to create a detailed pixel-level deformation map.

**Figure 7:**
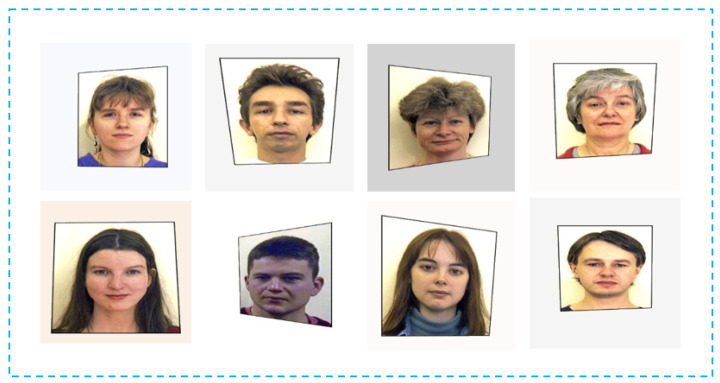
Samples from the synthetic dataset.

For a task like image deformation, obtaining an extensive dataset may not be feasible. To handle this problem, data augmentation techniques can be used to increase the diversity of training data. Initially, a range of colors is used to create various background textures, selected to closely match the color of a sheet of paper under different lighting scenarios. Variations in illumination conditions and image color are introduced through jitter applied in the HSV (Hue, Saturation, Value) color space. Subsequently, a projective transformation is applied to simulate changes in viewpoint. In our experiment, data augmentation allows the model to learn from a wider range of scenarios. This, in turn, improves the network’s ability to generalize to unseen data and handle variations present in real-world images. Figure 7 shows examples of images in the synthetic dataset.

#### 3.2.3. Training Details

We synthesized 100k distorted images with their corresponding forward mapping as ground truth; 90% of our dataset is used for training and the rest for validation. Input images are resized to 256 × 256 before being transmitted through the network. This allows the network to efficiently process images of different resolutions and helps alleviate computational and memory demands associated with deep networks while maintaining sufficient resolution for training. The NAdam optimizer is used with a learning rate of 0.0002. The training consists of 100 epochs without a dynamic learning rate schedule, and eight samples are fed to the network in each batch. The loss is defined as a combination of three different functions as follows:

The Mean Squared Error loss ($loss_{m}$):

(4) $$loss_{m}=\frac{1}{N}\sum_{i=1}^{N}{\left(y_{i}-{\mathrm{label}}_{i}\right)}^{2}$$

where *N* represents the number of samples, $y_{i}$ denotes the predicted value at index *i* and ${\mathrm{label}}_{i}$ is the corresponding ground truth value.

The feature loss ($loss_{f}$) that combines absolute differences and a regularization term:

(5) $$loss_{f}=\frac{1}{N}\sum_{i=1}^{N}\left|y_{i}-{\mathrm{label}}_{i}\right|-0.1\times \left|\frac{1}{N}\sum_{i=1}^{N}\left(y_{i}-{\mathrm{label}}_{i}\right)\right|$$

The background loss ($loss_{b}$) to penalizes negative values in *y*:

(6) $$loss_{b}=\frac{1}{N}\sum_{i=1}^{N}\min\left(y_{i},0\right)$$

Finally, the total loss (L), obtained as the sum of the previous individual losses, is used to train and evaluate our model:

(7) $$L=loss_{m}+loss_{f}+loss_{b}$$

Table 1 shows the training parameters for the primary components of Cam-Unet architecture.

**Figure 8:**
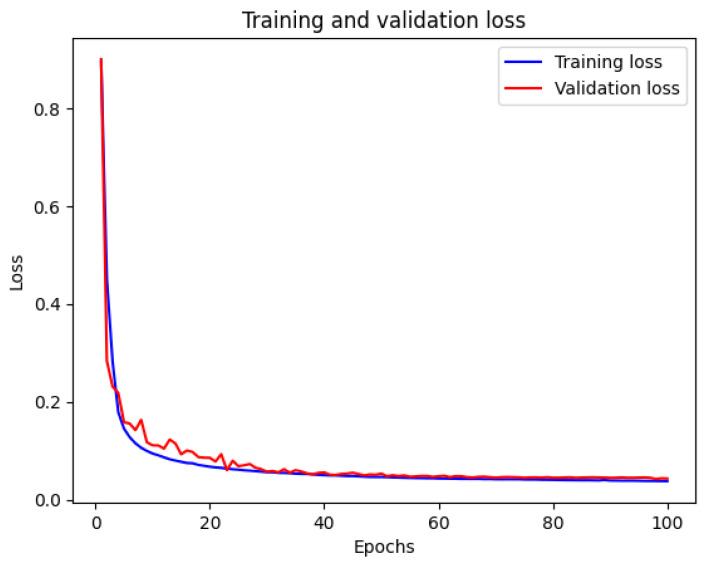
Training and validation loss.

The training and validation loss curves for the Cam-Unet model are shown in Figure 8. As illustrated, losses decrease over epochs, reaching stability after the hundredth epoch. The validation loss curve closely tracks the training loss, showing strong generalization capabilities of the model to unseen data. The close alignment between training and validation curves indicates the model’s understanding of the mapping task, leading to efficient convergence.

## 4. Experimental Results

In this section, the proposed watermarking method is compared with two other methods [26,27] tailored for print-cam watermarking in ID images. These methods all share a common foundation: They employ the Fourier-based method [12] for watermark embedding, and they integrate correction techniques to handle perspective distortions associated with the print-cam process. Gourrame et al. [26] used a Hough lines transform-based method for perspective rectification, while Boujerfaoui et al. [27] proposed a recursive neural network to address distortions. Notably, a 180-bit watermark is embedded into the experimental images, while maintaining an average PSNR of 40 dB.

We present two tests: First, we apply simulated perspective distortions to ID watermarked images. Then, we conduct real print-cam attacks on the printed versions of the ID watermarked images, capturing them using two smartphones (Redmi Note 10 and Galaxy S21) in a freehand manner.

### 4.1. Simulation Print-Cam Test

**Figure 9:**
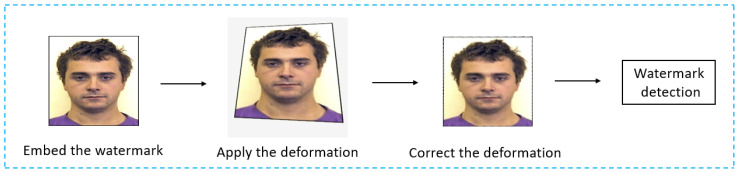
Simulated test procedure.

Here, we watermarked 500 ID images sourced from the PICS [47] database. Simulated print-cam attacks were executed and perspective correction was subsequently applied. All comparative methods were performed following uniform conditions and protocols. The testing procedure is illustrated in Figure 9.

**Figure 10:**
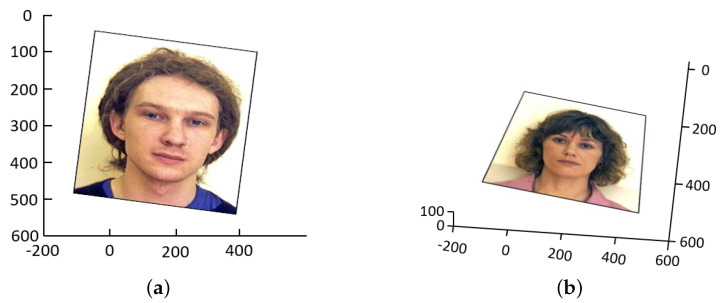
(**a**) 3D rotation (5°, −2°, 10°) with viewpoint (0°, 90°). (**b**) 3D rotation (5°, −2°, 10°) with viewpoint (10°, 60°).

To simulate perspective distortions, we apply a combination of 3D rotation to the image across the *x*, *y*, and *z* axes, along with variations in the viewpoints of the camera, defined by polar angles θ and ϕ. These angles determine the viewpoint relative to the scene, with θ representing the polar angle in the x−y plane and ϕ denoting the angle below or above the x−y plane. This simulation is intended to generate the typical distortions observed in images taken freehandedly with a smartphone. Hence, we randomly select rotation values for the *x* and *y* axes in the range −5° to 5°, while for the *z*-axis we choose between −10° and 10°. Similarly, the viewpoint values of θ and ϕ are randomly chosen within the ranges [0°, 10°] and [60°, 90°], respectively. The next figure (Figure 10) shows samples of simulated perspective distortions.

**Figure 11:**
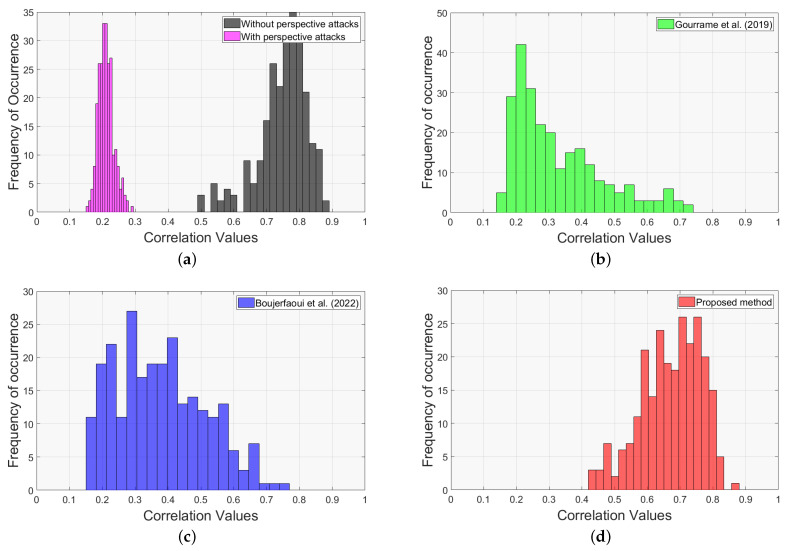
Empirical probability density functions of correlation values after perspective attacks rectification. (**a**) illustrates the ideal scenario (shown in black) in which no perspective attacks are present, and the worst case under perspective attacks (shown in pink). (**b**–**d**) illustrate the correlation results of the proposed method and methods in [26,27].

The detection results of all methods are shown in Figure 11, presented as histograms of empirical probability density functions of the correlation values. The frequency of occurrence denotes the number of times each correlation value appears within the dataset. In this context, the term correlation specifically refers to the maximum normalized cross-correlation, as defined in Equation (2).

The proposed watermarking system demonstrated superior performance, achieving an average correlation of 0.67, which significantly outperformed the other methods [26] (0.33) and [27] (0.38). Remarkably, the use of Cam-Unet for perspective correction results in a detection quality that is almost indistinguishable from scenarios in which no attack was present, unlike other perspective correction techniques. This distinction can be attributed to the limitations of the other perspective corrections, where residual rotations and translations can still survive even after corrective measures have been applied. Thus, these findings confirm the effectiveness of our watermarking approach compared with other methods. They also underline how well Cam-Unet addresses perspective distortions in the print-cam process.

To confirm these initial results, experiments were conducted in real-world scenarios, and the outcomes are reported in the subsequent section.

### 4.2. Real Print-Cam Test

**Figure 12:**
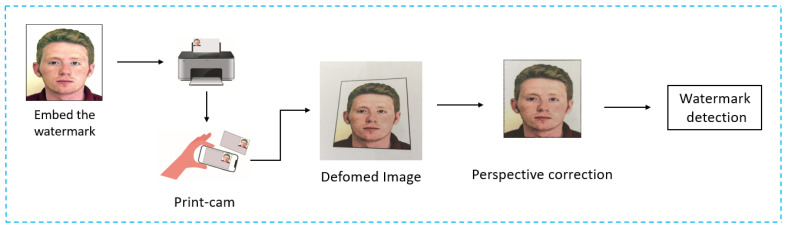
The process of the real-world test.

In this test, the three methods are evaluated under real print-cam conditions. We printed 500 ID images (250 watermarked and 250 non-watermarked) on paper using a Konica Minolta bizhub 450i printer. Each printed image measured 44 × 44 mm. Then, we captured these images using two different mobile devices; “Redmi Note 10” and “Galaxy S21”, with resolutions of 64 megapixels and 108 megapixels respectively. The image acquisition process was carried out in a freehand manner, under daylight illumination and without the use of filters or flash. The process of the test is illustrated in Figure 12.

**Figure 13:**
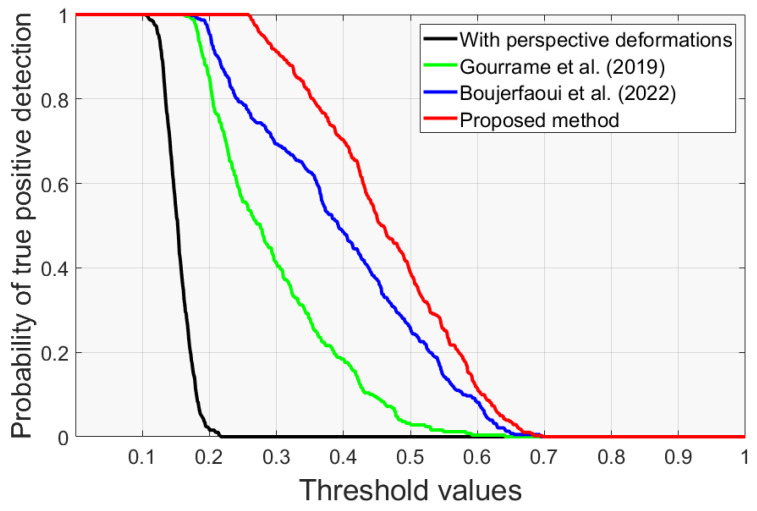
Probability of true positive detection as a function of the threshold values. The black line represents the worst-case scenario when perspective attacks are present. The other three lines represent the results of the proposed method and the other methods in [26,27].

After perspective corrections, the true positive detection is computed as a function of the detection threshold, with the results displayed in Figure 13.

The proposed watermarking system demonstrates a significant improvement in detection accuracy, reaching 90% at a higher threshold of 0.3 and outperforming the other methods [26] (40%) and [27] (70%) at the same threshold. The significant improvement can be attributed to the incorporation of the new correction technique, Cam-Unet, which effectively manages perspective distortions induced by the print-cam process. As a result, the proposed approach demonstrates superior performance compared to the other methods.

After that, the proposed Fourier watermarking scheme is evaluated in comparison to the other two methods using ROC curves.

**Figure 14:**
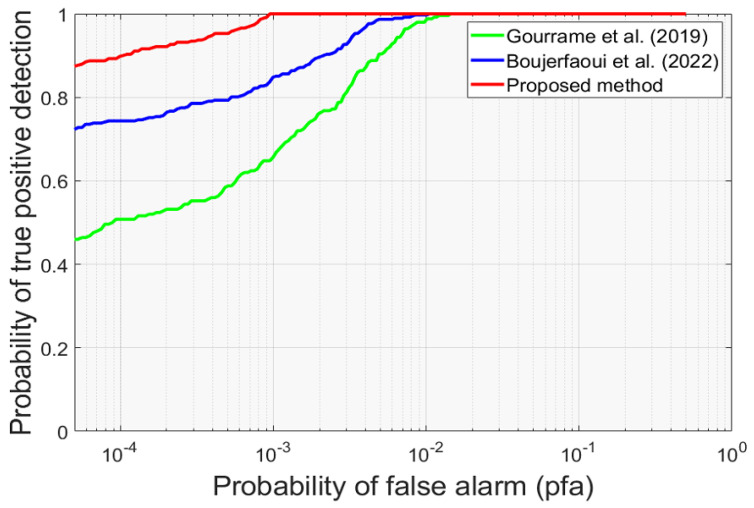
ROC curves comparison of the proposed method and methods in [26,27].

According to Figure 14, the proposed watermarking method excels with an exceptional detection rate of 90%, even at lower Probability of False Alarm (PFA) values. This performance represents a significant gap compared to other methods evaluated. These remarkable results underscore the substantial positive impact of the proposed correction method in mitigating perspective distortions during the print cam process. Furthermore, these outcomes validate the results obtained during simulated tests, reinforcing the reliability and practical applicability of the proposed method. Therefore, the consistent performance across both simulated and real-life situations underlines the effectiveness of the method in practical contexts.

Table 2 shows the minimum error rates observed for the three different methods. The proposed method stands out with a minimum error rate of 0.34%, while the other two methods [26,27] showed higher error rates of 1.83% and 1.05%, respectively. Given the industry standard of a 1% error rate, our method effectively meets this requirement. These results highlight the superiority of our proposed method, presenting a significantly lower error rate than existing approaches. This underlines its potential for practical applications on different smartphone models.

## 5. Conclusions

In this paper, we present a Fourier transform-based zero-bit image watermarking method, specially designed to withstand print-cam attacks and can be implemented on smartphones. The process includes Cam-Unet, a novel pre-processing network designed to correct projective deformations commonly encountered in freehand-captured images. This network is the first end-to-end neural network to directly remove perspective distortions, specifically tailored for the print-cam process. In an industrial context, notably concerning the security of ID images, experimental results demonstrate the superiority of the proposed method over competing techniques, presenting a remarkably low error rate of 0.34% compared to the significantly higher error rate of 1.83% observed with other methods. This highlights the real-world utility and effectiveness of our approach in protecting against print-cam attacks, known for their potential to severely distort images. The prototype presented here, developed through research and development (R&D), represents a significant advancement in watermarking technology specifically designed to address challenges posed by the print-cam process. However, it is crucial to acknowledge certain limitations inherent in our study. Print-cam variables such as lighting conditions, and paper quality can influence the results. Additionally, our experimental data may not cover all possible scenarios due to the diversity of technologies. Therefore, future research should aim to address a wide range of printers and smartphones to better understand the nuances of the print-cam process.

## Figures and Tables

**Table 1:** Training parameters.

Component	Number of Parameters (M)
Encoder 1	4.7
ASPP 1	0.9
Decoder 1	2.9
Encoder 2	1.1
ASPP 2	0.4
Decoder 2	3.6
**Total training parameters.**	13.6

**Table 2:** Minimal error rates of the three methods.

Method	Gourrame et al. [26]	Boujerfaoui et al. [27]	Proposed Method
**Minimal error rate**	1.83%	1.05%	0.34%

## Data Availability

The PICS dataset used in this study is available at https://pics.stir.ac.uk, accessed on 23 January 2022.
